# The Influence of Language on Spatial Reasoning: Reading Habits Modulate the Formulation of Conclusions and the Integration of Premises

**DOI:** 10.3389/fpsyg.2021.654266

**Published:** 2021-05-17

**Authors:** Thomas Castelain, Jean-Baptiste Van der Henst

**Affiliations:** ^1^Center for Cognitive Sciences, University of Neuchâtel, Neuchâtel, Switzerland; ^2^Trajectoires Team, Centre de Recherche en Neurosciences de Lyon, CNRS UMR 5292, Inserm UMR-S 1028, Université Lyon 1, Lyon, France

**Keywords:** mental models, reading habits, relational reasoning, spatial reasoning, mental scanning

## Abstract

In the present study, we explore how reading habits (e.g., reading from left to right in French or reading from right to left in Arabic) influence the *scanning* and the *construction* of mental models in spatial reasoning. For instance, when participants are given a problem like A is to the left of B; B is to the left of C, what is the relation between A and C? They are assumed to construct the model: A B C. If reading habits influence the scanning process, then readers of French should inspect models from left to right, whereas readers of Arabic should inspect them from right to left. The prediction following this analysis is that readers of French should be more inclined to produce “left” conclusions (i.e., A is to the left of C), whereas readers of Arabic should be more inclined to produce “right” conclusions (i.e., C is to the right of A). Furthermore, one may expect that readers of French show a greater ease in constructing models following a left-to-right direction than models following a right-to-left direction, whereas an opposite pattern might be expected for readers of Arabic. We tested these predictions in two experiments involving French and Yemeni participants. Experiment 1 investigated the formulation of conclusions from spatial premises, and Experiment 2, which was based on non-linguistic stimuli, examined the time required to construct mental models from left to right and from right to left. Our results show clear differences between the two groups. As expected, the French sample showed a strong left-to-right bias, but the Yemeni sample did not show the reverse bias. Results are discussed in terms of cultural influences and universal mechanisms.

## Introduction

### A Cultural Hypothesis in the Manipulation of Mental Models

Imagine that a grocer is telling you how fruits are arranged in the store:

Pears are to the left of Apples.
Oranges are to the right of Apples.
Lemons are in front of Pears.
Kiwis are in front of Oranges.

To make sense of these statements, you will probably build a mental model that is analogous to the situation they describe (DeSoto et al., 1965; Huttenlocher, 1968; Johnson-Laird, 1983):


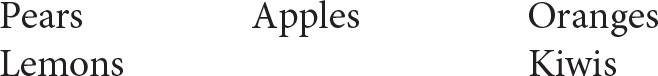


From this model, you may then infer relations that were not explicitly stated in the description, such as the relation between Lemons and Kiwis, namely, you may infer that “*Lemons are to the left of Kiwis*,” or equivalently, that “*Kiwis are to the right of Lemons*.” Although, these two conclusions describe the same situation, they may result from distinct mental processes. In an earlier study, Van der Henst and Schaeken (2005) argued that the formulation of conclusions may be driven by the scanning direction of mental models. In particular, if reasoners scan their model from left to right and formulate a conclusion while they perform the scanning, the item located on the left of the model will be the first to be encountered. The grammatical subject of the conclusion will thus likely refer to that item (i.e., Lemons are to the left of Kiwis). Alternatively, if reasoners scan their model from right to left, the grammatical subject of the conclusion is likely to refer to the item on the right of the mental model (i.e., Kiwis are to the right of Lemons).


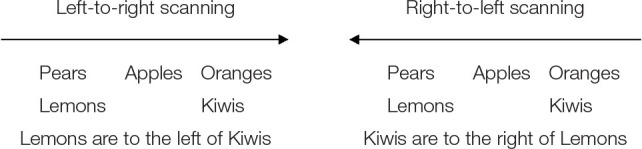


As the structure of mental models is not verbal but is analogous to the structure of the situations they represent, mechanisms involved in visuospatial processing are likely to contribute to the construction and manipulation of mental models (Vandierendonck and De Vooght, 1997; Knauff et al., 2002; Prado et al., 2010). Van der Henst and Schaeken (2005) proposed that a key factor that determines the scanning direction of spatial mental models is that of writing and reading habits, such as left-to-right (LTR) reading habits in Latin and Cyrillic, or right-to-left (RTL) reading habits in Arabic and Hebrew. Reading habits largely contribute to cultural differences in many visuospatial tasks and in more cognitive tasks that engage visuospatial mechanisms. Indeed, they are known to influence spatial attention (Spalek and Hammad, 2005), line estimation (Chokron and Imbert, 1993; Chokron and de Agostini, 1995; Chokron et al., 1998; Singh et al., 2000), image perception (Afsari et al., 2016), drawing orientation (Nachshon, 1985; Vaid et al., 2002; Kebbe and Vinter, 2013), aesthetic judgment (Vaid and Singh, 1989; Eviatar, 1997; Nachshon et al., 1999; Chokron and de Agostini, 2000; Pérez González, 2012; Chahboun et al., 2017), product choice (Ariga, 2018), memorization and recall of information (Nachshon et al., 1977; Padakannaya et al., 2002; Chan and Bergen, 2005), and number representation (SNARC effect, Dehaene et al., 1993; Zebian, 2005; Shaki and Fischer, 2008; Shaki et al., 2009; Shaki and Gevers, 2011), as well as the representation of time and events (Tversky et al., 1991; Maass and Russo, 2003; Chan and Bergen, 2005; Dobel et al., 2007, 2014; Fuhrman and Boroditsky, 2010; Ouellet et al., 2010).

### Evidence of the Influence of Reading Habits on Relational Reasoning

In their study dedicated to relational reasoning, Van der Henst and Schaeken (2005) asked a group of students who were readers of Dutch to answer questions from spatial descriptions similar to the one presented above and found that those students were more likely to formulate conclusions compatible with their LTR reading habits. Because spatial influences on mental representations provide strong evidence for the existence of mental models (Van der Henst, 2002; Van der Henst and Schaeken, 2005; Prado et al., 2008; von Hecker et al., 2016), the effect of reading habits on the representation of spatial descriptions and spatial reasoning has since been assumed in several studies. For instance, Jahn et al. (2007) found that people reading a reasoning premise like “A table is between a TV and a chair” more often represented its meaning with a mental model where the TV was on the left of the table and the chair was on its right (TV—table—chair), reflecting an LTR preference, than a model with a reverse arrangement (chair—table—TV), reflecting an RTL preference. However, the studies of Van der Henst and Schaeken (2005) and Jahn et al. (2007) included only readers of LTR languages, which did not allow for testing the prediction of an intercultural difference.

In later work, however, Román et al. (2013) did compare readers of an LTR language (i.e., Spanish) to readers of an RTL language (i.e., Moroccan Arabic) on the influence of model construction. They asked participants to draw situations described by auditory sentences, such as “A table is between a TV and a chair,” and observed a clear influence of reading habits. While in the Spanish group 70.7% of the drawings reflected an LTR representation (TV—table—chair), in the Moroccan group 61.7% of the drawings reflected an RTL representation (chair—table—TV); moreover, a group exposed to both RTL and LTR languages showed a weaker lateral bias. Research has also shown that the LTR bias in model construction could be reduced by exploring a speechless comic with RTL directionality (Román et al., 2018) or by reading a short text in which the direction of letters is reversed and which imposes an RTL reading (Román et al., 2015).

Reading habits also affect the evaluation process of conclusions that can be drawn from mental models. In this respect, von Hecker et al. (2016) found evidence of a lateral anchoring when reasoners construct a mental model from premises conveying a social linear order such as A > B, B > C, C > D, D > E (where “>” could mean richer, smarter, stronger, etc.), or E < D, D < C, C < B, B < A (where “ < ” could mean less rich, less smart, less strong, etc.). In their study, readers of English evaluated more quickly a pair when the socially dominant item of that pair was presented on the left side of the screen (e.g., B–D, if B is more dominant than D) than on the right side (e.g., D–B). This result suggests that the most dominant item of a linear order (A–B–C–D–E) was anchored on the left side of their mental models. However, for a group of non-university participants who were readers of an RTL language (i.e., Farsi), the effect was reversed, which indicated that these participants anchored the most dominant item on the right side of their mental model (von Hecker et al., 2016).

### The Current Study

Taken together, these studies show that people prefer to build certain models rather than others and that these preferences are guided by reading habits. In the current research, we extend these findings in two ways. First, we investigate the influence of reading habits on the scanning of mental models once they are built. We hypothesize that reading habits may induce different perspectives on the exploration and thus on the description of mental models. In Experiment 1, we test the prediction that LTR readers scan their model from left to right, whereas RTL readers scan their models from right to left. In line with Van der Henst and Schaeken (2005) proposal, we presume that the formulation of conclusions is likely to reveal the scanning directions. Hence, we assume that conclusions such as “X is to the left of Y” (henceforth “left” conclusions) should reveal an LTR scanning, whereas conclusions such as “Y is to the right of X” (henceforth “right” conclusions) should reveal an RTL scanning.

Second, while past research examined which model is preferentially built, we examine the influence of reading habits when the content of two premises is integrated into a single mental model. Indeed, the integration of premises is a crucial component of the building process (see Bonnefond et al., 2014). We thus compared the effort required to integrate premises in an LTR direction to the effort required to integrate premises in an RTL direction:


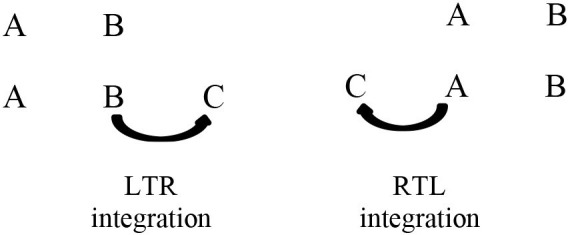


In order to dispose of two populations with opposite reading and writing systems and with relatively few influences from other reading habits, the two experiments were carried out with French (LTR) and Yemeni (RTL) participants. All the data were collected in 2006 at the University of Sana'a (Yemen) and 2007 at the Lumière University Lyon 2 (France). In Experiment 1, French participants should produce more “left” than “right” conclusions while the inverse pattern is expected for the Yemeni. In Experiment 2, the integration of the second premise should be facilitated when the problem requires a construction that matches with the reading habits of the participants. So, French participants should exhibit lower latencies in the LTR mode of construction compared to the RTL construction, whereas the reverse pattern should be observed for the Yemeni.

## Experiment 1

### Participants

Forty-five French (39 females, mean_age_ = 19.4 years, SD = 2.46 years) and 46 Yemeni (43 females, mean_age_ = 20.1 years, SD = 2.55 years) undergraduate students participated in this experiment. None of the French students were Arabic speakers, and none of the Yemeni were French speakers (still some of them had very limited knowledge of English or French). Two participants from the French group were excluded from the analyses because none of their answers were correct. Four participants from the Yemeni group were also discounted, 2 because none of their answers were correct, one because she gave only “in front of”–type responses to all the problems, and 1 because she gave no answers at all.

### Methods

#### Materials

Participants had to solve eight 1-model problems (Table 1) as in Van der Henst and Schaeken (2005). Each problem consisted of a set of four premises and one question. Each problem referred to a set of fruits or vegetables whose name started with different letters. We selected fruits and vegetables that were familiar to the participants and used them according to the country. The first two premises involved the relational expression “to the left of” or “to the right of,” and the last two premises involved the relational expression “in front of.” The four sentences were followed by a question asking the relation between the last two items introduced in the description. For each problem, there were two correct conclusions, a “left” conclusion (e.g., “The kiwi is *to the left of* the nectarine”) and a “right” conclusion (e.g., “The nectarine is *to the right of* the kiwi”).

**Table 1:** The eight problems used in Experiment 1 and their associated mental models (the question is: “What is the relation between D and E?”).

**Eight problems**							
Pb1^*^	Pb2^*^	Pb3^*^	Pb4^*^	Pb5^**^	Pb6^**^	Pb7^**^	Pb8^**^
A left B	A left B	B right A	B right A	A left B	A left B	B right A	B right A
B left C	C right B	B left C	C right B	C left A	A right C	C left A	A right C
D front A	D front A	D front A	D front A	D front B	D front B	D front B	D front B
E front C	E front C	E front C	E front C	E front C	E front C	E front C	E front C
**Corresponding mental models**							
Pb1^*^	Pb2^*^	Pb3^*^	Pb4^*^	Pb5^**^	Pb6^**^	Pb7^**^	Pb8^**^
A B C	A B C	A B C	A B C	C A B	C A B	C A B	C A B
D E	D E	D E	D E	E D	E D	E D	E D

*The symbols*

* * and*

** * characterize, respectively, type 1 and type 2 problems*.

For the statistical analysis regarding the wording of conclusions, we only take into account the correct answers. Thus, to ensure having the highest amount of data per participant, we restricted our study to one-model problems due to the fact that they are easier to solve than two-model problems (Byrne and Johnson-Laird, 1989). The use of the eight problems permitted to control for their formulation (the order of presentation, the spatial relation in the two first premises) and, most importantly in this study, the relative positions of the last two items that could impact the wording of conclusion (for the details, see Van der Henst and Schaeken, 2005). As reported in Table 1 (see corresponding mental models), problems 1–4 are characterized by the fact that D is located to the left of E, whereas problems 5–8 invite to construct a mental model where D is located to the right of E. In the rest of the article, we will refer to them as “type 1” (problems 1–4) and “type 2” (problems 5–8) problems.

#### Procedure

Participants were tested in group in a classroom of their University. The instructions and the eight problems were presented in a written format in a nine-page booklet. The first page contained the instructions and an example. Each problem was presented separately on a sheet, and the presentation order of the problems was pseudo-randomized between participants. Participants were instructed not to draw or take notes and to provide only one answer for each problem. The problems were translated from French to Arabic by a native speaker and reviewed by a second native informant. All the responses provided by the Yemeni participants were translated to French by a native speaker.

### Results and Discussion

#### Data Treatment and Statistical Methods

Data processing, analyses, and plotting were conducted with R in RStudio (RStudio Team, 2020) using the following packages: tidyverse (Wickham, 2017), lme4 (Bates et al., 2015), sjPlot (Lüdecke, 2020a), sjlabelled (Lüdecke, 2020b), sjmisc (Lüdecke, 2018), coefplot (Lander, 2018), and Lattice (Sarkar, 2008).

We used generalized linear mixed-effects models (GLMMs, binomial family, logit link) to assess the effects of problem type (type 1 coded as−1 and type 2 coded as 1) as within-subjects factor and language (Arabic coded as−1 and French coded as 1) as between-subjects factor on accuracy (0 for incorrect answer and 1 for correct answer) and on the wording of conclusions (0 for “left” conclusion and 1 for “right” conclusion). These contrasts were coded as orthogonal custom contrasts (i.e., planned comparisons) in which the fixed effects estimated the differences between conditions, and the intercept estimated the grand mean of dependent variables. All the GLMMs included the intercept of participants and items as crossed random effects, and the models were fitted via the maximum likelihood estimation procedure.

A step-up strategy was used to implement our models. We started with the implementation of a null model, which included the intercept as a fixed effect, and the intercept of items and persons as crossed-random effects. Then, we successively included the fixed effects and the interaction. Finally, we used two criteria to select the winning model: the log-likelihood statistic along with Akaike Information Criterion (AIC). The model with the lowest AIC was preferred. To disambiguate the model selection process, we included the AIC differences (i.e., ΔAIC)^1^. According to Burnham and Anderson (2002, p. 70), values of ΔAIC between 0 and 2 indicate little support to discriminate between models, ΔAIC from 4 to 7 indicate less support for the model with higher AIC, and ΔAIC > 10 suggests no support for the model with the higher AIC.

#### Accuracy

**Figure 1:**
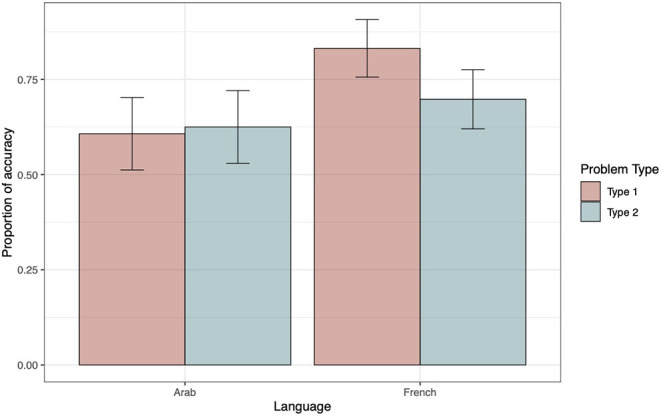
Accuracy as a function of Problem type and Language. The within-subjects 95% confidence intervals were computed following the method proposed by Morey (2008) and were implemented using the R functions developed by Chang (2018).

Table 2 presents the ΔAIC values for each model for accuracy (see top panel). The ΔAIC values exclusively support model 5, which was regarded as the winning model. The negative estimate of the interaction term between language and problem type indicates that, for type 1 problems, the French outperformed the Arabic speakers (see estimate parameters, top panel of Table 3). To better understand the interaction, we performed a main-effects analysis of model 5. To this end, three contrasts were computed: French type 1 problems vs. French type 2 problems, Arab type 1 problems vs. French type 2 problems, and Arab type 2 problems vs. French type 2 problems (see top panel of Table 4). The first contrast was the only one to reveal an effect on accuracy, and its positive estimate suggests that French participants were more accurate when confronted to type 1 than type 2 problems. Figure 1 helps illustrate these findings.

**Table 2:** Models testing sequence for Accuracy and Wording of conclusions.

**No**.	**Fixed effects**	***Df***	**AIC**	**ΔAIC**	**Log-lik**
**Accuracy**					
1	Int.	3	820.16	12.9610	−407.08
2	Int., Language	4	811.90	4.6978	−401.95
3	Int., Problem type	4	820.14	12.9366	−406.07
4	Int., Language, Problem type	5	811.87	4.6707	−400.94
5	Int., Language × Problem type	6	807.20	0.0000	−397.60
**Wording of conclusions**					
1	Int.	3	753.67	14.6943	−373.84
2	Int., Language	4	751.15	12.1755	−371.58
3	Int., Problem type	4	744.12	5.1422	−368.06
4	Int., Language, Problem type	5	741.63	2.6498	−365.81
5	Int., Language × Problem type	6	738.98	0.0000	−363.49

*Language, French and Arab; Problem type, type 1 and type 2; Df, degree of freedom; AIC, Akaike Information Criterion; ΔAIC, differences between the winning model and remaining models; Log-lik, log-likelihood*.

**Table 3:** Parameter estimates of models Accuracy and Wording of conclusions.

**Effect**	**Parameter**	**Estimate**	**95% CI**	**Standard error**	***Z* (*p*-value)**
Accuracy^*^					
Intercept	Intercept_._	0.97301	[0.70, 1.26]	0.14440	6.738 (<0.001)
Slope of Language	French–Arab	0.42915	[0.20, 0.69]	0.12859	3.337 (<0.001)
Slope of Problem type	Type 2–Type 1	−0.19266	[−0.41, 0.01]	0.10750	−1.792 (0.07)
Interaction of Language × Problem type	French–Arab × Type 2–Type 1	−0.23544	[−0.44, −0.06]	0.09133	−2.578 (<0.01)
Wording of conclusions^**^					
Intercept	Intercept_._	−0.18932	[−0.46, 0.09]	0.14840	−1.276 (0.20)
Slope of Language	French–Arab	−0.28620	[−0.53, −0.02]	0.13737	−2.083 (<0.05)
Slope of Problem type	Type 2–Type 1	0.58915	[0.37, 0.83]	0.11527	5.111 (<0.001)
Interaction of Language × Problem type	French–Arab × Type 2–Type 1	0.20944	[0.013, 0.41]	0.09788	2.140 (<0.05)

*Language, Arab and French; Problem type, type 1 and type 2; Arab–French, contrasts (i.e., differences) between Arabic and French speakers; type 2–type 1, contrasts (i.e., differences) between type 2 and type 1 problems*.

* *Random effects for the Accuracy model. Random intercept for subjects = 0.81 (standard deviation), 95% confidence interval [CI] = 0.42, 1.06; random intercept for items = 0.16 (standard deviation), 95% CI = 0.00, 0.32*.

** *Random effects for the Wording of conclusions model. Random intercept for subjects = 0.90 (standard deviation), 95% CI = 0.53, 1.22; random intercept for items = 0.16 (standard deviation), 95% CI = 0.00, 0.32*.

**Table 4:** Parameters estimates of the interaction of model 5 for Accuracy and the interaction of model 5 for Wording of conclusions.

**Effect**	**Parameter**	**Estimate**	**95% CI**	**Standard error**	***Z* (*p*-value)**
Interaction model 5 Accuracy^*^					
Intercept	Intercept	0.9741	[0.52, 1.44]	0.2345	4.154 (<0.001)
Slope of French type 1 and type 2 problems	French Type1–FrenchType2	0.8562	[0.26, 1.45]	0.2997	2.857 (<0.01)
Slope of Language type 1 and type 2 problems	Arab Type1–FrenchType2	−0.4730	[−1.17, 0.15]	0.3226	−1.466 (0.14)
Slope of Language type 2 problems	ArabType2–FrenchType2	−0.3874	[−0.97, 0.19]	0.3024	−1.281 (0.20)
Interaction model 5 Wording of conclusions^**^					
Intercept	Intercept_._	−1.3015	[−1.83, −0.82]	0.2702	−4.808 (<0.001)
Slope of French type 1 and type 2 problems	FrenchType2–FrenchType1	1.6332	[1.02, 2.31]	0.3188	5.123 (<0.001)
Slope of Language type 1 problems	ArabType1–FrenchType1	−0.2731	[−1.10, 0.51]	0.4049	−0.674 (0.5)
Slope of Language type 1 and type 2 problems	ArabType2–FrenchType1	3.2013	[2.38, 4.19]	0.4453	7.189 (<0.001)

*Language, Arab and French; Problem type, type 1 and type 2; FrenchType1–FrenchType2 or FrenchType2–FrenchType1, contrasts between type 2 and type 1 problems for French speakers; ArabType1–FrenchType2, contrasts between Arabic speakers for type 1 problems and French speakers for type 2 problems; ArabType2–FrenchType2, contrasts between Arabic and French speakers for type 2 problems; ArabType1–FrenchType1, contrasts between Arabic and French speakers for type 1 problems; ArabType2–FrenchType1, contrasts between Arabic speakers for type 2 problems and French speakers for type 1 problems*.

* *Random effects for the Accuracy model. Random intercept for subjects = 0.81 (standard deviation), 95% confidence interval [CI] = 0.45, 1.08; random intercept for items = 0.16 (standard deviation), 95% CI = 0.00, 0.35*.

** *Random effects for the Wording of conclusions model. Random intercept for subjects = 0.97 (standard deviation), 95% CI = 0.50, 1.31; random intercept for items = 0.00 (standard deviation), 95% CI = 0.00, 0.33*.

#### Wording of Conclusions

**Figure 2:**
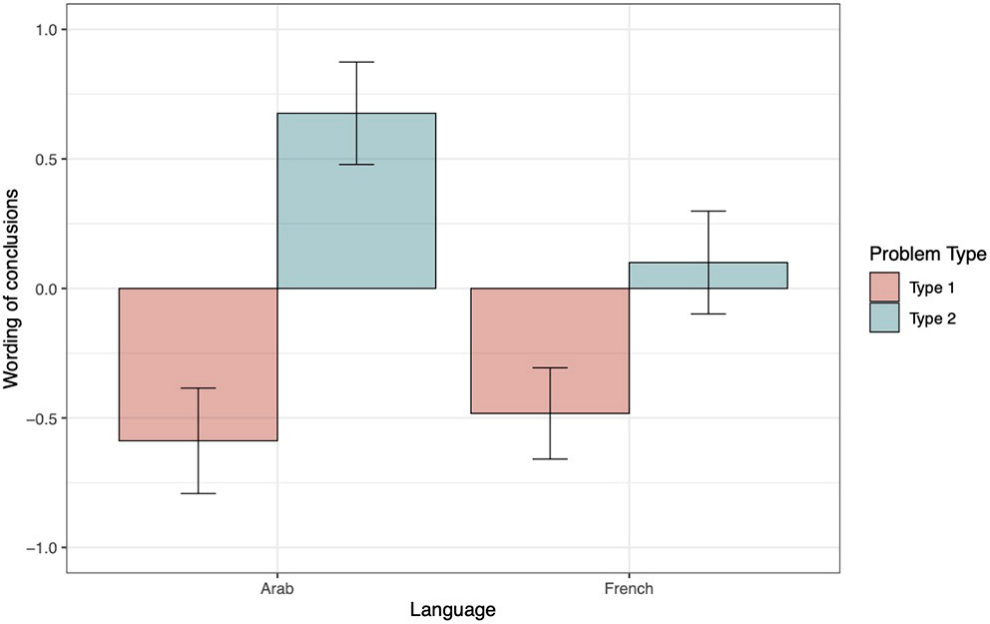
Wording of conclusions as a function of Problem type and Language. A negative score indicates a higher proportion of “left” conclusions (coded as −1), and a positive score, a higher proportion of “right” conclusions (coded as +1). The within-subjects 95% confidence intervals were computed following the method proposed by Morey (2008) and were implemented using the R functions developed by Chang (2018).

To analyze the wording of conclusions, we only took into account correct responses. On the whole, the French tend to produce more “left” (61%) than “right” (39%) conclusions [*t*_(262)_ = −3.59, *p* <0.001], whereas Yemeni participants produced as many “left” (47%) as “right” (53%) conclusions [*t*_(206)_ = 0.79, *p* = 0.43]. The bottom panel in Table 2 displays the model testing sequence for wording of conclusions as a function of language and problem type. The ΔAIC values exclusively favor model 5, which was considered as the winning model. The positive estimate of the interaction term between language and problem type indicates that, for type 2 problems, Yemeni produced more “right” conclusions than French participants (see estimate parameters, bottom panel of Table 3). Again, to break down the interaction, we performed a main-effects analysis using French type 1 problems as a reference. The contrast between French and Arabic speakers for type 1 problems revealed that both groups generated as many “left” conclusions (see bottom panel of Table 4). Figure 2 illustrates these findings.

#### Discussion

The first experiment revealed that reading habits influenced the way participants scanned their mental models and described the conclusions they drew from these models. On the whole, French participants produced more “left” than “right” conclusions regardless of problem type, replicating the results obtained with readers of Dutch by Van der Henst and Schaeken (2005). On the contrary, Yemeni participants produced as many “left” as “right” conclusions. This overall group difference was qualified by the type of problems. When dealing with type 1 problems, which induce an LTR scanning, French and Yemeni participants produced more “left” conclusions (respectively, 74 and 79%). However, with type 2 problems, which invite an RTL scanning, Yemeni showed a clear preference for “right” (84%) over “left” conclusions (16%), whereas French produced as many “left” as “right” conclusions (45 vs. 55%). The formulation of conclusions drawn by Yemeni was more dependent on the direction of model construction prompted by problem type than on their reading habits. This suggests that Yemeni participants were less influenced by the writing direction of their native language than French. Complementary to our main results, we observed a difference in terms of accuracy between our two groups of participants. This effect could be attributed to the fact that Yemeni participants were less familiar with psychological experiments than the French students (for similar results, see von Hecker et al., 2016).

## Experiment 2

### Participants

Twenty-two French (12 females, mean_age_ = 21.7 years, SD = 2.03 years) and 33 Yemeni (16 females, mean_age_ = 22.1 years, SD = 2.26 years) undergraduate students participated in this experiment. None of the French students were Arabic speakers, and none of the Yemeni were French speakers (but some of them had very limited knowledge of English or French).

### Methods

#### Materials

**Figure 3:**
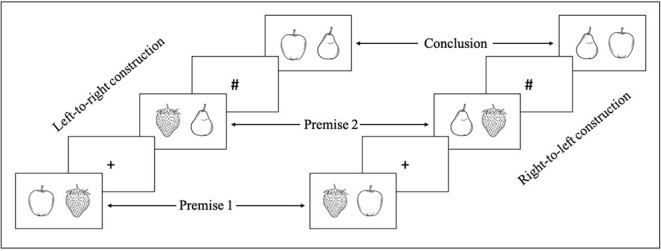
Illustration of the sequence of events and the two directions of construction.

Each problem was formed by two premises reporting a spatial relation between three objects (e.g., The apple is to the left of the lemon; the lemon is to the left of the orange) and a conclusion to evaluate (e.g., The apple is to the left of the orange). In order to limit the influence of linguistic factors in the task, the problems involved photographs of real items instead of sentences (Figure 3). Items were selected according to two criteria: First, they were available in both countries, and second, they had a symmetrical shape in order to avoid any influence of orientation. For each problem, the three items belong to the same group, namely, either fruits, vegetables, or kitchen utensils. Sixteen triplets of stimuli, therefore 16 different problems, constitute the main material of our experiment (Figure 3).

**Figure 4:**
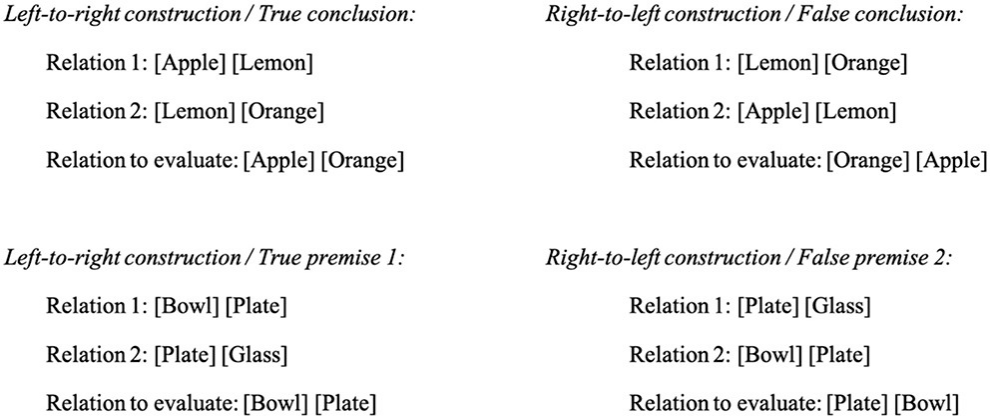
Illustration of the four types of conclusions.

There were two types of problems that differed according to the direction of model construction (LTR vs. RTL, see Figure 3), and there were two types of conclusions (true vs. false, see Figure 4). The conclusions proposed to the participants could take four different forms: (a) a relation that can be inferred from the two premises (true conclusion); (b) a relation that contradicts the relation inferred from the premises (false conclusion); (c) a relation that has already been presented (true premise 1 or 2); and (d) a relation that contradicts a relation that has been presented (false premise 1 or 2). Conditions (c) and (d) were introduced to prevent the use of a short-cut strategy that would consist in solving the task without constructing any mental model. Indeed, one could solely remember the position of one of the two elements of the first (or second) premise (e.g., [Apple] in Figure 3) and verify if it is present at the same location in the conclusion, without making any inference or integrating the two premises. The fact that participants were not able to predict which relation they would be asked to evaluate forced them to build a mental model containing the three objects (Figure 4).

#### Procedure

Before starting the reasoning tasks, the experimenter exposed to the participants the four types of problems they will have to solve (Figure 4), and they were familiarized with the task by solving eight training problems. The instructions were translated from French to Arabic by a native speaker and reviewed by a second Yemeni informant.

Participants had to solve 32 problems presented in two blocks. Each block contained eight problems in which the conclusion involved one item of each premise and eight problems in which the conclusion involved two items from a single premise. Half of the problems required an LTR construction, and the other half required an RTL construction. From one block to the other, the same triplets of stimuli were used, but they were presented in the opposite direction (e.g., a problem presented from LTR in the first block was presented from RTL in the second block). The order of presentation of the problems was randomized across participants. Stimuli were presented on a laptop using E-Prime 1.1 (Schneider et al., 2002). Participants were instructed to answer as fast and as accurate as possible and instructed (and reminded before each block) to keep their fingers on the keyboard throughout the entire experiment.

Each relation of the problems was presented separately (Figure 3). The trial begins with the first premise. To move from the first premise to the second premise, and then to the conclusion, participants had to press the spacebar. A cross (+) was presented in the center of the screen before the second premise, and a pound sign (#) before the conclusion. Participants were then instructed to give their answer (regarding the validity of the conclusion) using one of the two keys (e.g., “true” or “false”) signaled by a Post-it (respectively, green or red) on the keyboard (positions counterbalanced between subjects).

### Results and Discussion

#### Data Treatment and Statistical Methods

The same statistical method and criteria employed for Experiment 1 were adapted to analyze the data collected in Experiment 2. We first used GLMMs (binomial family, logit link) to assess the effect of direction of construction (LTR coded as−1 and RTL coded as 1) as within-subjects factor and language (Arab coded as−1 and French coded as 1) as between-subjects factor on the accuracy (0 for incorrect answer and 1 for correct answer). We then fitted linear mixed models to examine the effects of the same variables (using the same contrasts coding scheme) on the processing time of premise 2 (RT premise 2). RTs premise 2 of each population lower than the fifth percentile and higher than the mean +2.5^*^SD were excluded from the analyses, and the resulting data set underwent a log transformation before performing the analyses.

#### Accuracy

**Figure 5:**
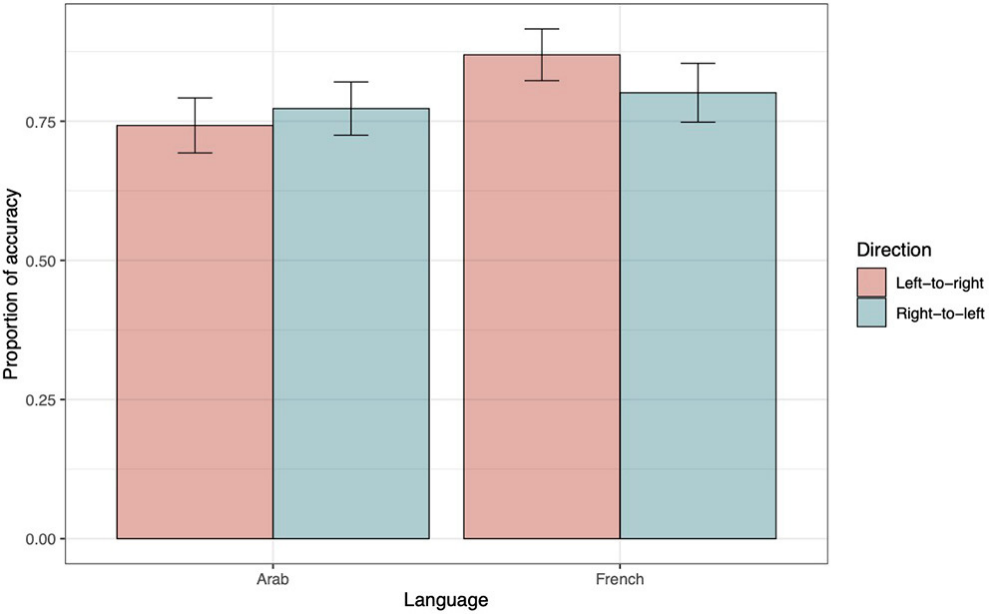
Accuracy as a function of Direction and Language. The within-subjects 95% confidence intervals were computed following the method proposed by Morey (2008) and were implemented using the R functions developed by Chang (2018).

The ΔAIC values exclusively support model 5, which was thus taken as the winning model (see top panel of Table 5). The positive estimate of the interaction term between language and direction indicates that, in the LTR condition, the French outperformed the Arabic speakers (see estimate parameters, top panel of Table 6). Nevertheless, in order to fully explore the interaction, we performed a main-effects analysis using French RTL as a reference (see top panel of Table 7). The only difference appeared for French participants when the LTR and the RTL were contrasted. Its positive estimate indicates a higher accuracy in the former compared to the latter condition. Figure 5 illustrates these findings.

**Table 5:** Models testing sequence for Accuracy and Premise 2 processing time.

**#**	**Fixed effects**	***Df***	**AIC**	**ΔAIC**	**Log-lik**
**Accuracy**					
1	Int.	3	1,683.6	6.928	−838.81
2	Int., Language	4	1,681.2	4.5044	−836.58
3	Int., Direction	4	1,685.4	8.7104	−838.68
4	Int., Language, Direction	5	1,682.9	6.2521	−836.45
5	Int., Language × Direction	6	1,676.7	0.0000	−832.33
**Premise 2 processing time**					
1	Int.	4	1,648.2	40.733	−820.11
2	Int., Language	5	1,649.5	41.952	−819.72
3	Int., Direction	5	1,636.6	29.151	−813.32
4	Int., Language, Direction	6	1,637.9	30.422	−812.96
5	Int., Language × Direction	7	1,697.5	0.000	−796.75

*Language, French and Arab; Direction, left-to-right and right-to-left; Df, degree of freedom; AIC, Akaike Information Criterion; ΔAIC, differences between the winning model and remaining models; Log-lik, log-likelihood*.

**Table 6:** Parameters estimates of models Accuracy and Premise 2 processing time.

**Effect**	**Parameter**	**Estimate**	**95% CI**	**Standard error**	***Z* (*p*-value)**
Model accuracy^*^					
Intercept	Intercept_._	1.71968	[1.38, 2.10]	0.17802	9.660 (<0.001)
Slope of Language	French–Arab	0.35815	[0.04, 0.70]	0.16336	2.192 (<0.05)
Slope of Direction	LTR–RTL	0.09991	[−0.03, 0.23]	0.06684	1.495 (0.13)
Interaction of Language × Direction	French–Arab × LTR–RTL	0.19364	[0.06, 0.33]	0.06688	2.895 (<0.01)
Model premise 2 processing time^**^					
Intercept	Intercept_._	7.94555	[7.82, 8.07]	0.06065	131.002 (<0.001)
Slope of Language	French–Arab	−0.04910	[−0.17, 0.07]	0.05961	−0.824 (0.41)
Slope of Direction	LTR–RTL	−0.05486	[−0.08, −0.03]	0.01185	−4.632 (<0.001)
Interaction of Language × Direction	French–Arab × LTR–RTL	−0.06783	[−0.09, −0.04]	0.01183	−5.732 (<0.001)

*Language, Arab and French; Direction, left-to-right (LTR) and right-to-left (RTL); French–Arab, contrasts (i.e., differences) between French and Arabic speakers; LTR–RTL, contrasts (i.e., differences) between left-to-right and right-to-left directions*.

* *Random effects for the Accuracy model. Random intercept for subjects = 1.04 (standard deviation), 95% confidence interval [CI] = 0.80, 1.37; random intercept for items = 0.25 (standard deviation), 95% CI = 0.05, 0.46*.

** *Random effects for the Processing time premise 2 model. Random intercept for subjects = 0.42 (standard deviation), 95% CI = 0.35, 0.52; random intercept for items = 0.04 (standard deviation), 95% CI = 0.00, 0.08*.

**Table 7:** Parameters estimates of the interaction of model 5 for Accuracy and the interaction of model 5 for Premise 2 processing time.

**Effect**	**Parameter**	**Estimate**	**95% CI**	**Standard error**	***Z* (*p*-value)**
Interaction model 5 accuracy^*^					
Intercept	Intercept	1.7843	[1.24, 2.38]	0.2832	6.300 (<0.001)
Slope of French right-to-left and left-to-right	FrenchLTR–FrenchRTL	0.5871	[0.15, 1.03]	0.2203	2.665 (<0.01)
Slope of Language right-to-left	ArabRTL–FrenchRTL	−0.3290	[−1.04, 0.36]	0.3473	−0.947 (0.34)
Slope of Language right-to-left and left-to-right	ArabLTR–FrenchRTL	−0.5165	[−1.23, 0.17]	0.3460	−1.493 (0.13)
Interaction model 5 premise 2 processing^**^					
Intercept	Intercept_._	8.01914	[7.83, 8.21]	0.09488	84.518 (<0.001)
Slope of French right-to-left and left-to-right	FrenchLTR–FrenchRTL	−0.24539	[−0.32, −0.17]	0.03612	−6.794 (<0.001)
Slope of Language right-to-left	ArabRTL–FrenchRTL	−0.03745	[−0.28, 0.20]	0.12166	−0.308 (0.76)
Slope of Language right-to-left and left-to-right	ArabLTR–FrenchRTL	−0.01152	[−0.25, 0.23]	0.12173	−0.095 (0.92)

*Language, Arab and French; Direction, left-to-right (LTR) and right-to-left (RTL); FrenchLTR–FrenchRTL, contrasts for French speakers between the left-to-right and the right-to-left directions; ArabRTL–FrenchRTL, contrasts between Arabic and French speakers for the right-to-left direction; ArabLTR–FrenchRTL, contrasts between Arabic speakers in the left-to-right direction and French speakers in the right-to-left direction*.

* *Random effects for the Accuracy model. Random intercept for subjects = 1.04 (standard deviation), 95% confidence interval [CI] = 0.80, 1.37; random intercept for items = 0.25 (standard deviation), 95% CI = 0.05, 0.46*.

** *Random effects for the Processing time premise 2 model. Random intercept for subjects = 0.42 (standard deviation), 95% CI = 0.35, 0.52; random intercept for items = 0.04 (standard deviation), 95% CI = 0.00, 0.08*.

#### Premise 2 Processing Time

**Figure 6:**
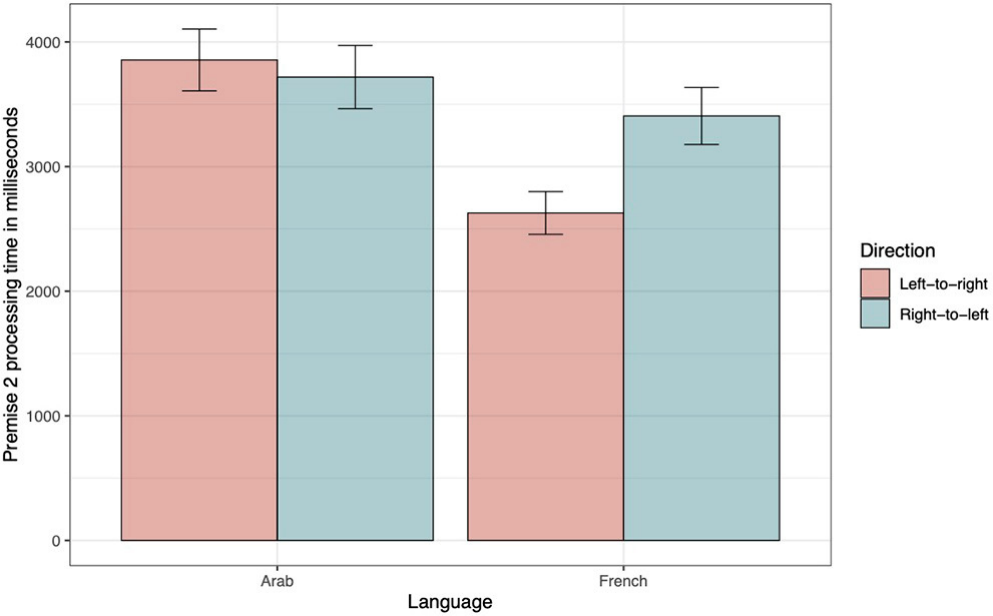
Premise 2 processing time as a function of Direction and Language. The within-subjects 95% confidence intervals were computed following the method proposed by Morey (2008) and were implemented using the R functions developed by Chang (2018).

In the following analyses, only accurate trials were included. The ΔAIC values exclusively favor model 5, which was thus considered as the winning model (see bottom panel of Table 5). The negative estimate of the interaction term between direction and language suggests that, in the LTR condition, the French were faster than the Arabic speakers to treat premise 2 (see bottom panel of Table 6). Again, we performed a main-effects analysis to break down the interaction based on the same contrasts as used previously (see bottom panel of Table 7). The contrast for French speakers revealed that they significantly took more time processing premise 2 in the RTL condition. Figure 6 illustrates these findings.

#### Discussion

Experiment 2 showed that reading habits affected how people construct their mental models. Indeed, the processing time of the second premise revealed a facilitating effect for French participants for LTR problems compared to RTL problems. Interestingly, such difference was not observed for Yemeni participants. As in Experiment 1, the Yemeni participants did not show an RTL preference, whereas the French participants show a clear preference for the LTR construction. Additionally to our main results, we noticed a difference in terms of response time between our two samples. Again, this difference could be attributed to the lack of familiarity of the Yemeni participants with psychological experiments but also with the use of computers, because at the time of the data collection, none of them possessed a personal or home computer (for similar results, see von Hecker et al., 2016).

## General Discussion

In this article, we report the results of two complementary experiments investigating the influence of reading habits on spatial reasoning. The results indicated that participants exposed to opposite reading habits differed in the way they reason. These data add up to the rich literature on reading habits showing that this cultural factor impacts a variety of cognitive processes, from low-level skills, such as perception or attention, to high-order cognition such as relational reasoning. In particular, they extend the results of earlier studies showing that reading habits determine the mental models that people preferentially construct (Jahn et al., 2007; Román et al., 2013, 2015, 2018; von Hecker et al., 2016). In the current study, French and Yemeni participants did not show the same pattern of results when describing conclusions from mental models and integrating information within a model. While French participants displayed a clear LTR bias in both experiments, there was not such a bias for Yemeni participants.

These results, however, contradict some of our predictions as Yemeni participants did not show an RTL bias. One explanation for this asymmetry is the possible exposure of Yemeni participants to LTR languages. They were University students and were thus likely to be exposed to LTR languages, through the influence of Western culture and the English language, whereas an exposure to RTL languages was probably less likely in the French group. Past research has shown that the directional bias reported in RTL readers is not always observed to the same extent as the directional bias found in LTR readers, even for low-level processes as visual scanning (Abed, 1991). In a variety of tasks such as picture naming and recall (Padakannaya et al., 2002), sentence–picture matching (Maass and Russo, 2003), or numerical mapping (Dehaene et al., 1993; Rashidi-Ranjbar et al., 2014; von Hecker et al., 2016), it was also found that the magnitude of this bias is inversely proportional to the duration of exposure to LTR languages. Moreover, a quick training or exposure to certain reading direction patterns seems to be enough to influence how people process information (Román et al., 2015, 2018; Afsari et al., 2016). Finally, it is also worth noting that in von Hecker et al. (2016) work, the student RTL sample did not show any directional bias in the mental model they constructed. It is only in the non-university RTL sample that a right-anchoring effect was reported, as these participants were faster at evaluating a relational pair when the socially dominant item of that pair was presented on the right of the screen (von Hecker et al., 2016). It may therefore be useful in future research to test non-student RTL populations to see if an RTL bias is likely to occur with both tasks of our study.

Another possible explanation of the asymmetry we found relies on the hemispheric specialization of the human brain. Data collected from split-brain patients highlighted the specialization of the right hemisphere for visuospatial processes (for a review, see Gazzaniga, 2005). Furthermore, according to the attentional model developed by Kinsbourne (1970), each hemisphere generates a horizontal attentional vector directed to the contralateral visual hemispace. The spatial nature of the task proposed to participants would engage predominantly the right hemisphere which attentional vector is directed from LTR, prompting them to inspect and construct mental models proceeding from left to right. Indeed, experimental evidence suggests that human beings are endowed with an LTR bias when dealing with spatial exploration or spatial representations. Although first reported in hemispatial negligent patients (for a review see, Corbetta and Shulman, 2011), this asymmetrical spatial bias has been reported in face perception among adults and children (Mertens et al., 1993; Leonards and Scott-Samuel, 2005; Guo et al., 2010), in line-bisection tasks (Bowers and Heilman, 1980), and in visual exploration of natural images (Calen Walshe and Nuthmann, 2014; Ossandón et al., 2014). This LTR bias seems to be an evolutionary inheritance as it has been reported not only in humans but also in rhesus monkeys and domestic dogs (Guo et al., 2009).

Another source of evidence in support of a universal LTR bias relies on the line of research developed by Chatterjee et al. (1995a,b, 1999). Based on the assumption that elementary spatial primitives precede linguistic encoding (Chatterjee, 2001), and generalizing the results obtained from an agrammatical patient, Chatterjee et al. (1999) sustain the hypothesis that events communicated orally are translated into spatial representations following an LTR trajectory. This directional bias would be the consequence of the overlap between the neuronal networks involved in the treatment of verbs and the spatial attention of the left hemisphere that is deployed according to an LTR vector (Chatterjee et al., 1999).

According to the last two accounts, a universal LTR bias would interact with reading habits (Maass and Russo, 2003) with the consequence of being potentialized for LTR readers while attenuated for populations with RTL reading and writing systems. Conducting similar experiments with illiterate participants or a population that do not possess a writing system would help to unravel the contribution of each factor and better measure the impact of a universal bias.

Still, the very fact that we observed different patterns in the two populations provides new evidence of the analogical nature of the representational processes involved in relational reasoning. Not only people create a spatial representation of the problem they are exposed to, but they also navigate into their mental models in order to generate inferences.

## Data Availability Statement

The datasets presented in this study can be found at Open Science Framework: 10.17605/OSF.IO/QXDF9.

## Ethics Statement

Ethical review and approval was not required for the study on human participants in accordance with the local legislation and institutional requirements. Written informed consent for participation was not required for this study in accordance with the national legislation and the institutional requirements.

## Author Contributions

TC: project administration, design, data collection, formal analysis, writing—original draft, writing—review, and editing. J-BV: design, writing—original draft, writing—review, and editing. All authors contributed to the article and approved the submitted version.

## Conflict of Interest

The authors declare that the research was conducted in the absence of any commercial or financial relationships that could be construed as a potential conflict of interest.

## Footnotes

^1^The procedure to compute the ΔAIC differences implicates to select the model with the lowest AIC, that is, preliminary, a winning model. Then, the differences between the preliminary winning model and the remaining models are computed. After obtaining these differences the winning model adopts a value of 0 and the other models adopt the differences.
